# Epidemiology of Pneumococcal Carriage among HIV-Infected Individuals in the Conjugate Vaccine Era: A Study in Southern Ghana

**DOI:** 10.1155/2019/3427174

**Published:** 2019-02-13

**Authors:** Nicholas TKD Dayie, Michael Baffuor-Asare, Appiah-Korang Labi, Noah Obeng-Nkrumah, Edeghonghon Olayemi, Margaret Lartey, Hans-Christian Slotved, Eric S. Donkor

**Affiliations:** ^1^Dept. of Medical Microbiology, School of Biomedical and Allied Health Sciences University of Ghana, Accra, Ghana; ^2^Dept. of Microbiology, Korle Bu Teaching Hospital, Accra, Ghana; ^3^Dept. of Medical Laboratory Science, School of Biomedical and Allied Health Sciences University of Ghana, Accra, Ghana; ^4^Dept. of Haematology, School of Biomedical and Allied Health Sciences University of Ghana, Accra, Ghana; ^5^Dept. of Medicine, School of Medicine and Dentistry, University of Ghana, Accra, Ghana; ^6^Dept. of Bacteria, Parasites and Fungi, Statens Serum Institut, Copenhagen, Denmark

## Abstract

Carriage of pneumococcus is considered as the precursor for development of pneumococcal disease. In sub-Saharan Africa, very little research has been done on the pneumococcus in relation to people with HIV infection in the era of pneumococcal conjugate vaccines. This study investigated pneumococcal carriage among HIV/AIDS patients in southern Ghana to determine the prevalence, risk factors, serotypes and antibiotic resistance of the organism. This was a cross sectional study involving 245 HIV/AIDS patients recruited from Korle Bu Teaching Hospital and Princess Marie Louis Hospital in Accra from November 2016 to March 2017. Epidemiological data on demographic, household and clinical features of the study participants were collected. Nasopharyngeal (NP) swabs were also collected from the study participants and cultured for* Streptococcus pneumoniae*; the isolates were serotyped by latex agglutination and Quellung reaction. Antimicrobial disc susceptibility was performed on the isolates, and antibiotics tested included tetracycline, erythromycin, cotrimoxazole, levofloxacin, oxacillin and ceftriaxone. Prevalence of pneumococcal carriage among the study participants was 11% (95% CI: 7.4 to 15.6); carriage among children and adults was 25% (95% CI: 14% to 38.9%) and 7.3% (95% CI: 4% to 11.9%) respectively. School attendance (p=0.001) and history of pneumococcal disease in the past year (p=0.001) were significantly associated with pneumococcal carriage. The most prevalent pneumococcal serotypes carried by the study participants were 19A (15.4%) and 23F (15.4%). Serotype coverage of the various pneumococcal vaccines were PCV10 (23.1%), PCV13 (42.3%) and PPV23 (50%). The prevalence of pneumococcal multidrug resistance was 18.5%. In conclusion, pneumococcal carriage among HIV-infected children was three-fold higher compared to carriage among HIV-infected adults. Pneumococcal carriage among both HIV-infected children and adults in the study area tends to be characterized by a predominance of non-vaccine serotypes and a considerable level of multidrug resistance.

## 1. Introduction

*Streptococcus pneumoniae* (pneumococcus) is part of the normal flora of the upper respiratory tract of humans but is also implicated in severe invasive diseases such as meningitis, septicaemia and pneumonia [1, 2]. Globally, the incidence of pneumococcal infections is about one million, majority of which occur in the developing world particularly in sub-Saharan Africa [3]. Compared to their healthy counterparts, HIV-infected children have about forty times greater risk of developing invasive pneumococcal disease [4]. The burden of pneumococcal disease is further complicated by the increasing resistance of the pneumococcus to a wide range of antibiotics [5]. High prevalence of multidrug resistant pneumococci carried by HIV-infected individuals have been reported [6, 7], and this could be partly due to the use of some antibiotics such as cotrimoxazole as prophylaxis in these patients. Thus the public health burden of pneumococcus tends to be much higher among HIV-infected individuals compared to healthy populations.

In sub-Saharan Africa, there is very little information on the pneumococcus in relation to HIV serostatus, as very few studies have been carried out on this subject. With the availability of Pneumococcal Conjugate Vaccines (PCVs), there is the need for relevant epidemiological data to be obtained from populations at-risk of pneumococcal disease, such as HIV-infected individuals. Pneumococcal conjugate vaccines are both well tolerated and immunogenic in HIV-infected infants and adults [8]. On the other hand, the pneumococcal polysaccharide vaccine (PPV23) appears to provide limited protection in immuno-compromised individuals, though this vaccine has wider serotype coverage than current PCVs [9, 10]. It is recommended that HIV-infected patients aged 19 years or older receive one dose of 13-valent Pneumococcal Conjugate Vaccine (PCV13) followed by a booster vaccination with PPV23 [10]. Carriage of pneumococcus is the precursor for development of invasive pneumococcal disease and PCVs are based on suppressing pneumococcal carriage, and hence incidence of the disease [9, 11]. Thus, carriage studies represent an appropriate model for providing vaccine related epidemiological data on the pneumococcus, as well as understanding the host-pathogen relationship of the organism. In Ghana, PCV13 was introduced in 2012 and is given to children at 6, 10 and 14 weeks of age. Though the vaccine could benefit HIV-infected older children and adults in Ghana, this has not been previously explored and the relevant pneumococcal epidemiological data in this regard is also lacking. In a previous study, we investigated the epidemiology of pneumococcal carriage among HIV-infected children in Ghana for the first time and reported several novel findings [12]. For example, pneumococcal serotype 16F, which had been rarely reported in Ghana, was one of the serotypes commonly carried by HIV-infected children [12]. Since our previous study was limited to children less than 13 years and there have been no other related studies in Ghana, the epidemiology of pneumococcal carriage in the population of HIV-infected individuals in the country has not been fully studied. To fill this research gap and confirm our previous findings on pneumococcal carriage in HIV-infected children, the current study was undertaken. In this study, we present pneumococcal carriage data on HIV-infected children and adults with the aim of describing the carriage prevalence, risk factors, serotypes and antibiotic resistance.

## 2. Materials and Methods

### 2.1. Study Site

The study was done at the Korle Bu Teaching Hospital (KBTH) and Princess Marie Louis Hospital (PML). The Korle Bu Teaching Hospital is the largest hospital in Ghana and is located in Accra, the capital city of the country. The Department of Medicine of KBTH has a 25-bed capacity unit for managing HIV/AIDS adult patients; the unit attends to about 250 HIV positive out patients on each clinic day. The PML hospital is the main paediatric hospital in Accra and has approximately 400 registered cases of HIV/AIDS patients. About 20 HIV-positive patients are seen on each clinic day at PML. Both KBTH and PML serve as referral hospitals in southern Ghana and are within one kilometre from the Department of Medical Microbiology of the School of Biomedical and Allied Health Sciences, University of Ghana where laboratory analysis of the specimens was carried out.

### 2.2. Study Design and Sampling

This was a cross-sectional study in which 245 consecutive HIV/AIDS patients were enrolled at the HIV clinics of the two hospitals from November 2016 to March 2017. Only HIV/AIDS patients in a steady state who came for their routine check-ups were included in the study. For the purposes of this study, steady state was defined as patients who were in relatively good health and had not been hospitalized in the past one month prior to the study period and were not on any medication, apart from those used routinely in the management of the disease condition such as multivitamins, cotrimoxazole prophylaxis for opportunistic infection and antiretroviral drugs. HIV/AIDS patients on admission were excluded from the study, as well as outpatients who had taken antibiotics in the past two weeks prior to sampling. Information on demographics and clinical history of the study participants was obtained using a structured questionnaire. Data on clinical history was extracted mostly from clinical records of the study participants and included CD4 counts, antiretroviral use, pneumococcal vaccinations, cotrimoxazole prophylaxis, and previous pneumococcal diseases. Demographic data were obtained through interview of the study participants and included age, sex, residential features and lifestyle characteristics such as smoking. Nasopharyngeal samples were collected from the study participants using nylon-tipped paediatric sized swabs (Microrheologics, Brescia, Italy) by a trained nurse according to the WHO recommended procedures for detecting pneumococcal carriage [13]. Briefly, the study participant's head was tipped slightly backwards to ensure an easy straight passage of the swab from the anterior section of the nostril to the posterior region. The swab was passed in such a manner that it was parallel to the floor of the nostrils. Anytime resistance was encountered during the movement of the swab, it was withdrawn and the other nostril tried. Once the swab reached the nasopharynx, it was allowed to sit for 5-10 seconds and rotated at 180° to allow the tip to be saturated. The swab was gently withdrawn and placed in labelled vial containing 1.5ml of skim milk-tryptone-glucose-glycerin (STGG) medium. A pair of scissors disinfected with 70% alcohol was used to aseptically cut a length of 4 cm from the handle of the swab and allowed to fall into the vial and screw-capped immediately. These nasopharyngeal samples were transported on ice to the laboratory within one to three hours after sampling.

### 2.3. Laboratory Analysis

#### 2.3.1. Cultural Isolation and Serotyping of S. pneumoniae

The samples were inoculated onto 5% sheep blood agar (made selective by the addition of 5*µ*g/ml of gentamycin) and incubated in 5-10% CO_2_ at 35±2°C for a period of 18-24 hours to enable isolation and characterization of* S. pneumoniae*. All alpha-haemolytic colonies were subjected to Gram staining, and optochin susceptibility testing; optochin susceptible isolates were identified as* S. pneumoniae* [13]. Where specimens could not be processed immediately, they were stored in -80°C until ready for processing where they were brought to room temperature of 25°C before processing.

Serotyping of pneumococcal isolates was performed by Pneumotest-Latex agglutination kit (SSI Diagnostica, Hillerød, Denmark) and the results were confirmed by the Quellung reaction using serotype specific antisera (SSI Diagnostica) [14]. All specimens were screened for multiple serotypes.

*Antibiotic Susceptibility Testing of S. pneumoniae*. The Kirby Bauer method was used to evaluate antibiotic susceptibility of* S. pneumoniae* isolates, and the antibiotics tested were tetracycline (30 *μ*g), erythromycin (15 *μ*g), cotrimoxazole (25 *μ*g), levofloxacin (5 *μ*g) and oxacillin (1 *μ*g). Briefly, pure pneumococcal colonies from a culture between 18-20 hours old were emulsified in peptone water to achieve a turbidity of 0.5% on the McFarland standard. A sterile cotton swab was dipped into the suspension and pressed against the side of the tube to remove excess suspension. The tip of the swab was then used to spread the bacterial load evenly on the surface of freshly prepared Muller Hinton Agar with 5% sheep blood and incubated in 5-10% of CO_2_ concentration for 20-24 hours at 37±2°C. Penicillin and ceftriaxone minimum inhibitory concentrations (MICs) were determined for all oxacillin non-susceptible isolates using MIC strips (Oxoid Company, UK). Results were interpreted using the Clinical and Laboratory Standards Institute (CLSI) guideline 2016 [15].

#### 2.3.2. Data Analysis

Data was entered onto Microsoft Excel spreadsheet and analysed using the SPSS software version 22. Descriptive statistics such as percentages and proportions were used to calculate the pneumococcal carriage prevalence per age groups. Univariable analyses were done to determine the relationship between pneumococcal carriage and demographic, clinical and household features of the study participants: chi-square test was used for categorical variables, and analysis of variance for numeric variables. Logistic regression model was used to analyse exposures associated with carriage. Theoretical coverage of various pneumococcal vaccines was estimated. Antibiogram of pneumococcal isolates including multidrug resistance were determined; multidrug resistance was defined as resistance to penicillin and two or more different classes of antimicrobial agents. All the statistical analyses were performed at a significance level of 5% and* p*-values < 0.05 were considered significant.

## 3. Results

### 3.1. Demographics and Household Features of the Study Subjects

The 245 HIV/AIDS patients recruited in the study had a mean age of 34.4years with a range of 0.6-74years; 40% (98) were recruited from PML and had a mean age of 7.2 years (paediatric sample), while 60% (147) were from KBTH and had a mean age of 45.1 years (adult sample). Relevant data on other demographic and household features of the study participants is reported in Table 1. In terms of sex, religion, and residence, study participants recruited from PML and KBTH had similar demographics. Overall, the majority of the study participants were females (77.2%), Christians (81.2%) and lived in compound houses (66.9%). The average number of people per household was 14. Among the children, 73.5% (72/98) were found to be actively schooling while 92.5% (136/147) of the adult population were employed. A proportion of 1.4% (2/147) of the adults smoked tobacco actively and 1.4% (2/147) were passive tobacco smokers.

### 3.2. Clinical Features of the Study Participants

Clinical features of the study participants including CD4 counts, ART use, cotrimoxazole prophylaxis, respiratory symptoms and previous pneumococcal disease did not show significant differences between PML and KBTH. Overall, the mean CD4^+^T cell count of the HIV/AIDS patients was 732.9cells/mm^3^, 95.9% (235) of them were on antiretroviral drugs while 54.3% (133) were on cotrimoxazole prophylaxis. A proportion of 85.7% (210) of the study participants had experienced respiratory symptoms in the last month prior to the study and catarrh (excessive discharge or build-up of mucous in the nose or throat, associated with inflammation of the mucous membrane) was the most common symptom (52.2%). Twenty four (9.8%) of the study participants had a history of pneumococcal disease in the one year prior to the sampling and otitis media was the most common disease. The cases of pneumococcal disease were based on culture, and this information was extracted from the clinical records of the study participants as explained in Section 2.2. Only 42.9% (42/98) of the children based on their age and the timing of the introduction of the PCV13 in Ghana, had been vaccinated during the study; for children less than 5 years, 89.2% (33/37) had received PCV13.

### 3.3. Carriage of Pneumococcus and Associated Risk Factors

Overall 27 of the 245 study participants carried pneumococcus, which translates to a carriage prevalence of 11% (95% CI: 7.4 to 15.6); carriage among children and adults were 25% (95% CI: 14% to 38.9%) and 7.3% (95% CI: 4% to 11.9%) respectively. Pneumococcal carriage was highest in the age group, 5-10 years (35.9%) and much lower in the study participants who were ≥51 years (Table 2). In the logistic regression analysis, pneumococcal carriage was significantly associated with school attendance (p=0.001) and history of pneumococcal diseases in the 12-months prior to sampling (p=0.001).

### 3.4. Pneumococcal Antibiotic Resistance

Seven (25.9%) of the 27 pneumococcal isolates which were non-susceptible to oxacillin by the Kirby Bauer method were deemed to be penicillin non-susceptible* S. pneumoniae *(PNSP) and were further subjected to penicillin G and ceftriaxone MIC tests. Minimum Inhibitory Concentration values of the PNSP isolates ranged from 0.03-2.0 ug/mL for penicillin G, and 0.004-2.0 ug/mL for ceftriaxone. Applying the CLSI breakpoint for oral penicillin, 2/7 of the PNSP showed intermediate resistance (MIC= 0.12-2.0 *μ*g/mL), 1/7 was totally resistant (MIC ≥ 2 *μ*g/mL), while 4/7 were susceptible (MIC ≤ 0.06 *μ*g/mL). However, when the CLSI breakpoint for parenteral penicillin was applied, all the PNSP isolates were susceptible (MIC ≤ 2). Based on the CLSI breakpoint for ceftriaxone, 1/7 of the PNSP isolates exhibited intermediate resistance (MIC= 1.0-2.0 *μ*g/mL), none was totally resistant (MIC ≥ 2 *μ*g/mL), while 6/7 were susceptible (MIC ≤ 0.5 *μ*g/mL). Prevalence of pneumococcal resistance to the other antibiotics tested was levofloxacin (0.0%), erythromycin (7.4%), tetracycline (66.7%) and cotrimoxazole (92.6%). The prevalence of multidrug resistance among the pneumococcal isolates was 18.5% (5/27), and the predominant antibiotic combination for multidrug resistance was penicillin, cotrimoxazole and tetracycline.

### 3.5. Pneumococcal Carriage Serotypes

Of the 27 pneumococci isolated from the study subjects, serotyping was done on 25 as 2 isolates had lost viability. Serotyping of the isolates yielded sixteen different pneumococcal serotypes, with serotypes 19A (15.4%) and 23F (15.4%) as the most prevalent (Table 3). There was one case of dual serotype carriage (3.8%) with serotypes 3 and 19A. Based on the serotype data, theoretical coverage of PCV10, PCV13 and PPV23 were 23.1%, 42.3% and 50% respectively.

## 4. Discussion

In this study, we investigated various aspects of the epidemiology of pneumococcal carriage including the carriage prevalence, risk factors, serotypes and antibiotic resistance among HIV-infected people ranging from 0.6 to 74years. The overall prevalence of pneumococcal carriage was 11% and carriage was lower among adults (7.3%) than children (25%), which is consistent with previous studies [16–18]. It is worthy to note that our study provides post-vaccination data as the pneumococcal vaccine was introduced in Ghana in 2012. By comparison, a similar post-vaccination study by Donkor* et al*. [12] on HIV-infected children <13 years in Ghana reported a carriage prevalence of 27%, which is similar to the prevalence (25%) we observed in the same age group. However, there is a wide disparity between pneumococcal carriage prevalence reported among children in our study and that of a similar study in Tanzania, which reported a prevalence of 80% among children <12 years [19]. This Tanzanian study was done before the introduction of PCVs into the country in 2013, which may partly explain the relatively high pneumococcal carriage compared to our study. In a Cambodian study, Kremery* et al*. [20] observed a significant reduction in pneumococcal carriage among HIV-infected children after pneumococcal vaccination. It is important to note that unlike the current study, in the Tanzanian study, polymerase chain reaction (PCR) for detection of* S. pneumoniae* was performed on culture negative samples. This may partly account for the huge disparity in pneumococcal carriage prevalence observed between the two studies.

The significant association of pneumococcal carriage with school attendance among children in the current study has been previously reported [3]. Pneumococcal carriage is relatively higher among young children and convergence at places such as schools facilitates transmission and persistence of carriage [2, 21]. We also observed a significant association between pneumococcal carriage and history of pneumococcal disease in the past year prior to sampling, which could be related to the fact that pneumococcal carriage is a precursor for pneumococcal disease [2]. Though our data seem to suggest that pneumococcal disease rather precedes carriage, the true situation is that it only provides evidence of prior carriage in HIV-infected individuals with previous invasive pneumococcal disease. This is because the study was cross sectional in design, and therefore provides evidence of association, rather than causation.

Much of the recent interest in the epidemiology of pneumococci involves tracing the spread of penicillin resistance. In Ghana, there is wide disparity in the prevalence of pneumococcal resistance to penicillin reported by different studies and prevalence as high as 68% had been reported [3, 22–26]. This disparity could be partly due to the fact that some of these studies were published before 2008, when the breakpoint for definitions of penicillin resistance was more inclusive. It is important to note that when the CLSI breakpoints for parenteral use of penicillin (non-meningitis) is applied, all the PNSP in this study are susceptible. This suggests that penicillin is suitable for treatment of pneumococcal infections in HIV/AIDS patients in Accra and probably Ghana. It is encouraging to also observe very low levels of pneumococcal resistance to cefotaxime and levofloxacin in this study. This could be partly due to these antibiotics being on the Ghanaian market for a relatively brief period of time and therefore not being used extensively. In contrast, antibiotics such as cotrimoxazole and tetracycline have been on the Ghanaian market for a long time, resulting in a high usage and therefore high increased resistance. In the case of cotrimoxazole, this could be partly due to the use for prophylaxis among HIV/AIDS patients in Ghana. It is important to note that increased resistance to cotrimoxazole and tetracycline has been reported for several other bacterial pathogens in Ghana, highlighting the need to discontinue these antibiotics for empirical treatment [27–32].

The pneumococcal serotype data in this study contrasts with that reported by Donkor* et al*. [18] on a sample of HIV-infected children in Ghana. The two major serotypes observed in the current study including serotypes 19A and 23F were not observed in the study of Donkor* et al*. [18]. On the other hand, serotype 19F, which was the most prevalent serotype in the study of Donkor* et al*. [18] was the least prevalent serotype in the current study. While the study of Donkor* et al*. reported multiple serotype carriage prevalence of 34.3% [18], in this study we observed a prevalence of 3.8%. These observations probably indicate a high diversity of the pneumococcal population structure in HIV-infected people in Ghana and further studies especially, longitudinal carriage surveys are needed to provide insights into this. In agreement with the study by Donkor* et al*. [18], we observed predominance of non-vaccine pneumococcal serotypes resulting in low serotype coverage of 23.1-50% for various vaccines. For PCVs, this could indicate the phenomenon of serotype replacement where non-vaccine types replace vaccine types as a result of vaccination. Serotype replacement in pneumococcal carriage is to be expected in populations immunised with PCVs [33–35].

There are a few limitations of the study. The first is the relatively small sample size of 245. Secondly, some of the study participants were on cotrimoxazole prophylaxis, which could have affected recovery of pneumococci from the study participants. Due to the limited pneumococci isolated from the HIV-infected children and adults in this study, it was not feasible statistically to compare pneumococcal serotypes and antibiotic resistance between the two populations. Also, we had no pneumococcal carriage data on HIV-infected individuals prior to PCV introduction in Ghana, for comparison.

In conclusion, pneumococcal carriage among HIV-infected children was three-fold higher compared to carriage among HIV-infected adults. Pneumococcal carriage among both HIV-infected children and adults in the study area tends to be characterized by a predominance of non-vaccine serotypes and a considerable level of multidrug resistance. The high prevalence of non-vaccine serotypes underscores the need for effective antibiotic therapy among the study population in the event that these serotypes are implicated in disease. Full penicillin resistance was rare among pneumococci carried by the study subjects, providing evidence for the use of this antibiotic to treat pneumococcal infections among the HIV-infected patients. Additionally, levofloxacin and cefotaxime are appropriate for treating pneumococcal infections among the HIV patients.

## Figures and Tables

**Table 1:** Demographic and household characteristics of the study participants.

**Variable**	**Princess Marie Children Hospital**	**Korle Bu Teaching Hospital**	**Overall**
	**N (**%**)**	**N (**%**)**	**N (**%**)**
**Sex**			
Male	40 (40.8)	27 (18.4)	67 (27.3)
Female	58 (59.2)	120 (81.6)	178 (72.7)
**Age (years)**			
<5	37 (37.8)	0 (0)	37 (15.1)
5--10	39 (39.8)	0 (0)	39 (15.9)
11--20	22 (22.4)	0 (0)	22 (9.0)
21--30	0 (0)	21 (14.3)	21 (8.6)
31--40	0 (0)	42 (28.6)	42 (17.1)
41--50	0 (0)	52 (35.4)	52 (21.2)
51--60	0 (0)	24 (16.3)	24 (9.8)
61--70	0 (0)	6 (4.1)	6 (2.4)
71--80	0 (0)	2 (1.3)	2 (0.8)
**Religion**			
Christian	79 (80.6)	120 (81.6)	199 (81.2)
Moslem	19 (19.4)	24 (16.3)	43 (17.6)
Others	0 (0)	3 (2.1)	3 (1.2)
**Resident type** ^**+**^			
Self-contained	28 (28.6)	53 (36.1)	81 (33.1)
Compound House	70 (71.4)	94 (63.9)	164 (66.9)
**Occupation**			
Children attending school	72 (73.5)	N/A	72 (73.5)
Children not in school	26 (26.5)	N/A	26 (26.5)
Employed Adults	N/A	136 (92.5)	136 (92.5)
Unemployed adults	N/A	11 (7.5)	11 (7.5)
**Smoking status**			
Active smoker	N/A	2 (1.4)	2 (1.4)
Passive smoker	N/A	2 (1.4)	2 (1.4)
Non smoker	N/A	143 (97.2)	143 (97.2)

N- indicates number of study participants; N/A- not applicable

^+^average number of people per household = 14.

**Table 2:** *Streptococcus pneumoniae* carriage in HIV/AIDS patients of different age categories.

**Age Category**	**Frequency/Total**	**Carriage Prevalence (**%**)**
<5years	7/37	18.9
5-10years	14/39	35.9
11-20years	5/22	22.7
21-30years	2/21	9.5
31-40years	4/42	9.5
41-50years	4/52	7.7
51-60years	1/24	4.2
61-70years	0/6	0
71-80years	0/2	0

**Table 3:** *Streptococcus pneumoniae* serotypes isolated from HIV/AIDS patients.

**Serotype**	**Number**	%	**Serotype included in vaccine**
3	1	3.8	PCV-13, PPV-23
6B	1	3.8	PCV-10, PCV-13, PPV-23
10A	2	7.7	PPV-23
11A	1	3.8	PPV-23
13	2	7.7	Non-vaccine serotype
15A	1	3.8	Non-vaccine serotype
17F	1	3.8	PPV-23
19A	4	15.4	PCV-13, PPV-23
19B	1	3.8	Non-vaccine serotype
19F	1	3.8	PCV-10, PCV-13, PPV-23
16F	2	7.7	Non-vaccine serotype
23B	1	3.8	Non-vaccine serotype
23F	4	15.4	PCV-10, PCV-13, PPV-23
24F	1	3.8	Non-vaccine serotype
28F	1	3.8	Non-vaccine serotype
33A	1	3.8	Non-vaccine serotype
Non-typeable	1	3.8	Not applicable

25 pneumococcal isolates were serotyped and there was one isolate with dual serotypes. Thus the total number of serotypes were 26.

## Data Availability

The epidemiological data used to support the findings of this study are available from the corresponding author upon request.
